# Early Trabecular Development in Human Vertebrae: Overproduction, Constructive Regression, and Refinement

**DOI:** 10.3389/fendo.2015.00067

**Published:** 2015-05-01

**Authors:** Frank Acquaah, Katharine A. Robson Brown, Farah Ahmed, Nathan Jeffery, Richard L. Abel

**Affiliations:** ^1^MSk Laboratory, Department of Surgery and Cancer, Faculty of Medicine, Imperial College London, London, UK; ^2^School of Medicine, King’s College London, London, UK; ^3^Department of Archaeology and Anthropology, University of Bristol, Bristol, UK; ^4^Department of Mineralogy, The Natural History Museum, London, UK; ^5^Department of Musculoskeletal Biology, Institute of Ageing and Chronic Disease, University of Liverpool, Liverpool, UK

**Keywords:** vertebra, trabecular, bone growth, development, ontogeny, microcomputed tomography

## Abstract

Early bone development may have a significant impact upon bone health in adulthood. Bone mineral density (BMD) and bone mass are important determinants of adult bone strength. However, several studies have shown that BMD and bone mass decrease after birth. If early development is important for strength, why does this reduction occur? To investigate this, more data characterizing gestational, infant, and childhood bone development are needed in order to compare with adults. The aim of this study is to document early vertebral trabecular bone development, a key fragility fracture site, and infer whether this period is important for adult bone mass and structure. A series of 120 vertebrae aged between 6 months gestation and 2.5 years were visualized using microcomputed tomography. Spherical volumes of interest were defined, thresholded, and measured using 3D bone analysis software (BoneJ, Quant3D). The findings showed that gestation was characterized by increasing bone volume fraction whilst infancy was defined by significant bone loss (≈2/3rds) and the appearance of a highly anisotropic trabecular structure with a predominantly inferior–superior direction. Childhood development progressed via selective thickening of some trabeculae and the loss of others; maintaining bone volume whilst creating a more anisotropic structure. Overall, the pattern of vertebral development is one of gestational overproduction followed by infant “sculpting” of bone tissue during the first year of life (perhaps in order to regulate mineral homeostasis or to adapt to loading environment) and then subsequent refinement during early childhood. Comparison of early bone developmental data in this study with adult bone volume values taken from the literature shows that the loss in bone mass that occurs during the first year of life is never fully recovered. Early development could therefore be important for developing bone strength, but through structural changes in trabecular microarchitecture rather than bone mass.

## Introduction

Gestation, infancy, and childhood are periods of rapid bone growth (increase in size) and development (change in shape). It is generally understood that these early periods of ontogeny may have a significant influence on bone strength and fracture risk during old age (1–4). Intuitively this makes sense because in old age, particularly after the menopause or andropause, bone tissue wastes away, but individuals who develop stronger bone before adulthood will withstand greater age-related loss of bone before a fragility fracture occurs. As yet though very little data have been published regarding development of the material and structural properties that contribute to strength. Further, the small amount of data that have been published to date provides conflicting evidence.

Material properties (i.e., bone mineral density, BMD) have received the most attention and several studies have emphasized the importance of early BMD for adult bone strength. For example, body mass at birth (5) and 1 year (6) was correlated with postmenopausal bone mineral content, which suggests that gestational and infant development may contribute to adult bone strength. However, BMD of long bones actually decreases by about 30% during the first months of life (7) and peak bone mass is not acquired until young adulthood (1–3). Hence, development of BMD after childhood, and even after skeletal maturity, might have more of an impact on bone health in old age.

Mass also contributes to strength, but the development of bone mass has received only moderate attention, in part because of the difficulty of obtaining and analyzing 3D models of bone [e.g., from microcomputed tomography (micro-CT) scans]. The most comprehensive studies of prenatal and infant development have examined the limb bones. During the gestational period (4–9 months), the bone volume fraction (BVF) in the humerus and femur remained constant (8) or increased (9, 10). In infancy, the femur (11) and tibia (12) exhibited a decrease in volume fraction between 0 and 2 years. Therefore, in line with the reports on BMD, the adult pattern bone mass probably develops only after infancy.

Strength is also dependent upon the structure of bone. It is not clear how the development of trabecular microarchitecture contributes to changes in bone mass because these studies used a variety of methods to visualize and measure the trabeculae, making the data difficult to compare. Glorieux et al. (9) and Salle et al. (10) both employed conventional 2D histological histomorphometric methods, sectioning femora under a microscope. Both studies reported that the thickness and number of trabeculae increased during gestation. In contrast, Reissis and Abel (8) applied digital histomorphometric techniques using relatively low-resolution (100 μm) micro-CT scans of femora. Following the conventional studies, trabecular thickness was reported to increase, but number decreased. Postnatally, Ryan and Krovitz (11) and Gosman and Ketcham (12) both analyzed micro-CT scans in 3D to analyze the femur and tibia, respectively. Both limb bones exhibited a decrease in trabecular number between 0 and 2 years, but the femur tended toward decreasing thickness whilst the tibia tended toward an increase. The divergent growth patterns of trabecular number in the femur and tibia could have a huge effect on strength because connectivity is an important determinant of bone strength.

Unfortunately, these studies of mass and microarchitecture provide a fragmented insight into trabecular development as changes in morphology have only been quantified for restricted periods of development. However, the published studies all suggest that human trabecular growth and development is both dynamic and sequential (12, 13–15). This appears to be the case for other species (16). Specifically, the studies published to date and described here indicate that parturition in particular is strongly associated with a reverse in growth trajectory from increasing to decreasing bone BMD and mass, and from a more connected to a less connected structure. The downstream effect of this reduction in bone mass in adulthood/old age is not clear because infant and adult trabecular structure has not been compared.

In order to address the question as to why BMD and mass decrease after birth, if early development is so important for bone strength, more data are required on early bone development, and in particular before and after parturition. Analysis of early growth and development in vertebral bone, a key fragility fracture site in diseases such as osteoporosis, may reveal just how much infancy contributes to adult bone mass. If it is the case that this is an important developmental period in regard to future adult bone health then perhaps it might be possible in future to identify children who are at risk of fragility related skeletal disorders in old age. The aim of this study is therefore to document early bone development from gestation and infancy through to childhood (2.5 years), and then consider whether these periods are important for building adult bone strength.

## Materials and Methods

The ontogeny of vertebral trabecular architecture, a key fracture site, was analyzed for the developmental period between 6 months prenatal and 2.5 years postnatal. Microarchitecture was visualized using micro-CT and measured using BoneJ (17), a plugin for ImageJ (National Institutes of Health, USA; http://rsb.info.nih.gov/ij/index.html) and Quant 3D (University of Texas).

### Sample and provenance

A total of 120 vertebrae from complete modern human vertebral columns (C1 to L5) were included in the study. These vertebral series were derived from a 19th century collection of juvenile skeletons of documented age at death held at the Royal College of Surgeons, London. The sample was selected to include three developmental periods: (i) gestational, 6 months – term; (ii) infant, term – 1.2 years; (iii) early childhood, 1.2–2.5 years. The validity of growth curves collected from past populations (including the specimens in this study) has been discussed elsewhere (8).

### Digital histomorphometry

Vertebral columns were scanned at 28 μm^3^ voxel size using a Nikon X-Tek HMX-ST 225 system (18). Homologous bone regions were extracted from individual vertebrae by aligning the bodies into a plane defined by three type-II anatomical landmarks (19): the left, right, and anterior apices of the superior cortical rim on the vertebral body. Alignment was followed by extraction of the largest possible spherical volumes of interest (VOI) about the centroid of the vertebral bodies. Trabecular tissue was segmented using a binary global threshold (20) based on the frequency distribution of the gray values (CT numbers) in the scan (21–23). The threshold value was placed at the trough between the characteristic peaks for air and bone.

Architectural measures were collected including trabecular: BVF (BV/TV); thickness (Tb.Th); number (Tb.N); connectivity density (Conn.D); structure model index (SMI), and degree of anisotropy (DA). The methods and algorithms are detailed elsewhere (17, 21, 24) but a general description follows here. BVF is the amount of bone per unit volume and was calculated by dividing the number of threshold white voxels by the total number of voxels in the VOI (17). Trabecular thickness (Tb.Th) was calculated by fitting the largest sphere possible within a trabecula. The diameter of this sphere represents the thickness of the trabecular element (17). Trabecular number (Tb.N) was measured using the mean intercept length (MIL) method and involved calculating the sum of intersections between trabecular bone and a matrix comprised of a grid of lines (11, 24). Similarly, connectivity density measured the number of connections between trabeculae per cubic millimeter (21). Connectivity density was calculated by dividing the Euler characteristic by the volume of the sphere (17). Structure model index (SMI) measured the proportion of “rod” and “plate”-shaped trabeculae. SMI values ranges from 0 for a structure made of “plate”-shaped trabeculae to 3 representing a primarily “rod”-shaped one. Structures with a mixture of plate and rod shapes are assigned a value between 0 and 3. Anisotropy measured the direction and size of the preferred orientation of trabeculae, calculated as a ratio between the maximum and minimum radii of the MIL ellipsoid (17, 25–27). A value of one reflects a completely anisotropic structure whilst zero reflects an isotropic one. Anisotropic trabecular bone is highly orientated along one direction and is typically highly organized. In comparison, isotropic trabecular bone is not highly orientated and may have a disorganized or randomly orientated structure (12, 28).

### Statistical analysis

Structural growth and development of vertebral trabecular bone was analyzed using a one-way ANOVA with Tukey’s *post hoc* carried out using PAST version 2.15 (29). Measurements from each vertebral column (24 bones) were combined into one value for each individual.

## Results

**Figure 1:**
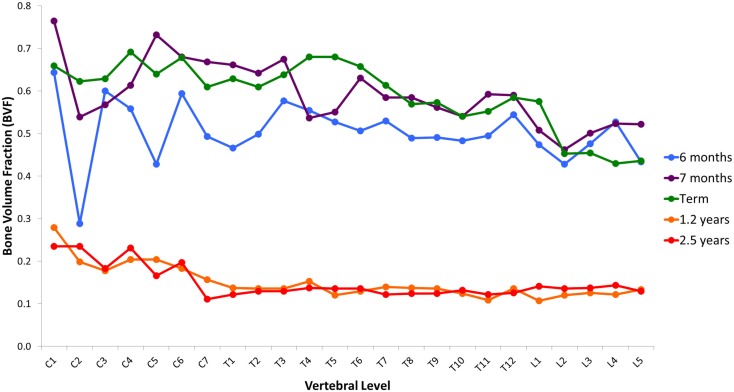
**Regional variation in vertebral trabecular bone volume fraction (BVF)**. Lumbar vertebrae exhibit significantly lower BVF in relation to cervical at 7 months, term, 1.2 years, and 2.5 years (one-way ANOVA *p* = 0.038). Values are from a single sample.

**Figure 2:**
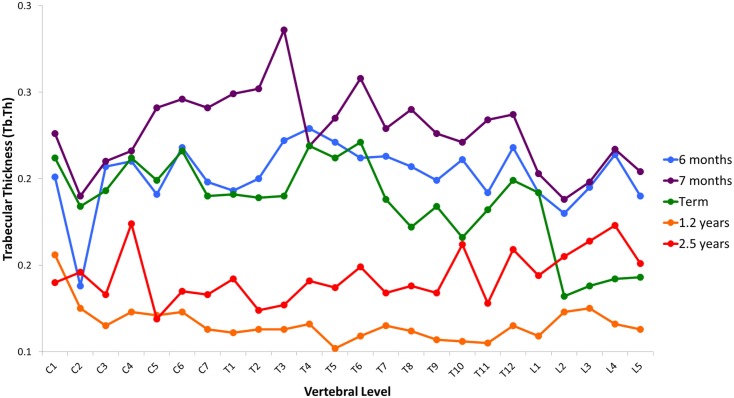
**Regional variation in trabecular thickness (Tb.Th)**. Cervical and thoracic vertebrae exhibit significantly thicker trabeculae than lumbar at 7 months and term (one-way ANOVA *p * = 0.050). Average values without error bars.

**Figure 3:**
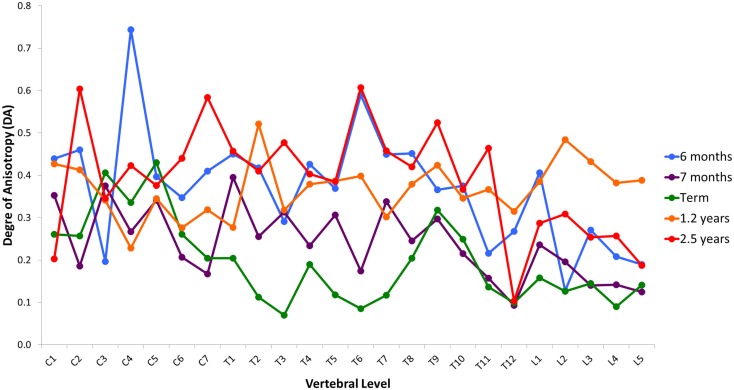
**Regional variation in degree of anisotropy (DA)**. Cervical and thoracic vertebrae exhibit significantly higher DA than lumbar at 6 months, 7 months and term, and 2.5 years (one-way ANOVA *p * = 0.001). Values are from a single sample.

**Figure 4:**
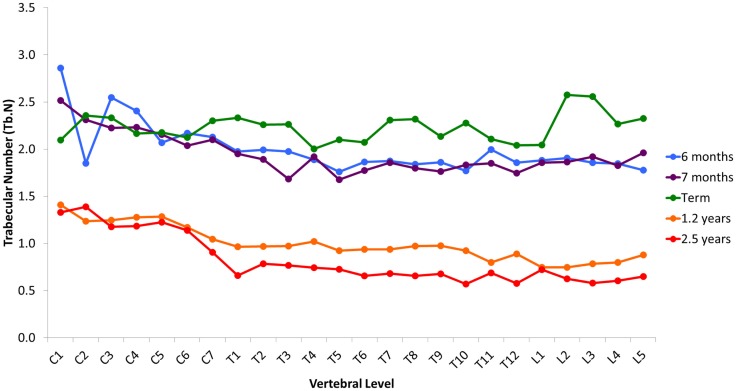
**Regional variation in vertebral trabecular number (Tb.N)**. Cervical vertebrae exhibit significantly more numerous trabeculae than lumbar at 6 months, 7 months, 1.2 years, and 2.5 years (one-way ANOVA *p * = 0.008). Values are from a single sample.

Variation in cancellous bone architecture with spinal level is presented in Figures 1–4. Lumbar vertebrae exhibit significantly lower BVF in relation to cervical at 7 months, term, 1.2 and 2.5 years (one-way ANOVA *p* = 0.038; Figure 1). Cervical and thoracic vertebrae exhibit significantly thicker trabeculae than lumbar at 7 months and term (one-way ANOVA *p* = 0.050; Figure 2). Cervical and thoracic vertebrae exhibit a significantly higher DA than lumbar at 6 months, 7 months and term, and 2.5 years (one-way ANOVA *p* = 0.001; Figure 3). Finally, cervical vertebrae exhibit significantly more numerous trabeculae than lumbar at 6 months, 7 months, 1.2 years, and 2.5 years (one-way ANOVA *p* = 0.008; Figure 4).

**Figure 5:**
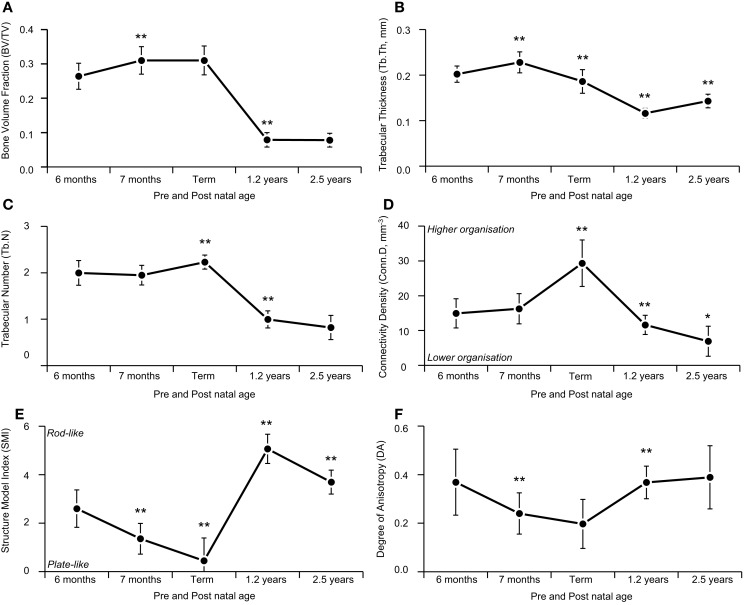
**Age-related variation in vertebral trabecular architecture**. Asterix denotes significant differences vs. previous age group (*p < 0.05; **p < 0.01). During gestation (6 months-term) there was a steady increase in BV/TV, peaking at term **(A)**. Increasing gestational BV/TV was attributable to increased Tb.N **(C)** which is also reflected by the increased Conn.D during this time period **(D)**. During infancy (term-1.2 years) vertebrae exhibited a significant decrease in BV/TV **(A)** due to a reduction in Tb.Th **(B)** and Tb.N **(C)**, causing trabecula to become more rod like [SMI **(E)**] and more anisotropic with a dominant inferior-superior axis [DA **(F)**]. During childhood (1.2–2.5 years) there is an increase in BV/TV **(A)** and trabecula return to a more, plate like shape [SMI **(E)**] while structure continues to become more anisotropic, organized along the inferior-superior axis [DA **(F)**].

While the maturational schedule of the regions of the spine appears to vary, the cervical, thoracic, and lumbar vertebrae all exhibited the same general pattern of change in trabecular microarchitecture (Table 1). Grouping the bones together, BVF increased prenatally (Figure 5A). The rise in BV/TV was significant between 6 and 7 months (*p * < 0.01) but not 7 and 9 months (*p * > 0.05). Increased volume was primarily attributed to growing trabecular thickness between 6 and 7 months (Figure 5B) followed by rising number between 7 and 9 months (Figure 5C). Elements grew thicker between 6 and 7 months (*p * < 0.01) then thinner from 7 to 9 months (*p * < 0.01). Whilst number decreased between 6 and 7 months (*p * > 0.05) then increased from 7 to 9 months (*p * < 0.01). Connectivity density increased between both 6 and 7 months (*p * > 0.05) and 7 and 9 months (*p * < 0.01; Figure 5D). In addition, there was a statistically significant shift from a rod-like to a plate-like structure between 6 and 7 months (*p * < 0.01) and between 7 and 9 months (*p * < 0.01; Figure 5E). Anisotropy decreased during gestational development, between both 6 and 7 months (*p * < 0.01) and 7 and 9 months (*p * > 0.05; Figure 5F).

**Table 1:** **Variation in trabecular microstructure in early development**.

Architectural measure	One way – ANOVA		Tukey’s *post hoc* between ages			
	*F*	*p*	6 vs 7	7 vs 9	9 vs 1.2	1.2 vs 2.5
Bone volume fraction	312	<0.001	0.001	1.000	0.001	1.000
Thickness	130	<0.001	0.001	0.001	0.001	0.001
Number	206	<0.001	0.935	0.001	0.001	0.052
Connectivity density	79	<0.001	0.860	0.001	0.001	0.006
Structure model index	162	<0.001	0.001	0.001	0.001	0.001
Anisotropy	16	<0.001	0.001	0.628	0.001	0.958

In infancy, there was a significant (2/3rds) decrease in BVF (*p * < 0.01, Figure 5A) via decreased trabecular thickness (*p * < 0.01, Figure 5B) and number (*p * < 0.01, Figure 5C). Connectivity density decreased (*p * < 0.01, Figure 5D) and there was a transition from plate-like to rod-like trabeculae (*p * < 0.01, Figure 5E), as well as a more highly orientated structure (*p * < 0.01, Figure 5F).

During early childhood, BVF remained constant (Figure 5A). Trabecular thickness increased (*p * < 0.01, Figure 5B) but number decreased (*p * > 0.05, Figure 5C). Connectivity density decreased (*p * < 0.01, Figure 5D). Trabeculae became more plate-like (*p * < 0.01, Figure 5E) and continued to become more highly orientated (*p * > 0.05, Figure 5F).

## Discussion

The ontogeny of early vertebral trabecular architecture during gestation, infancy, and early childhood was analyzed by spinal level, and then using a cross-sectional approach. The maturational schedule of the regions of the spine vary, with lumbar lagging slightly behind the thoracic region, but a similar pattern of microarchitectural change is common to all regions. Each cross-sectional developmental stage exhibited distinct patterns of microarchitectural growth and development. Before birth trabecular, BV/TV increased but became relatively isotropic. This pattern was reversed during infancy when BV/TV decreased but the structure became more anisotropic. In early childhood, a new pattern of development appeared, BV/TV remained constant but the DA continued to increase. Both the birth and the end of infancy may be associated with a marked change in growth trajectory of BV/TV.

### Patterns of bone modeling during early development

**Figure 6:**
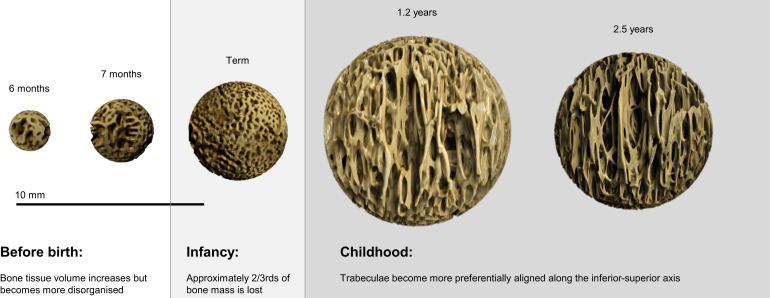
**Micro-CT reconstructions showing the development of vertebral trabecular microarchitecture of the first lumbar bone during the gestational, infant, and childhood periods**. The size of the bones has been scaled to demonstrate the variation in bone volume fraction.

Variation in the maturational schedule of vertebrae with spinal level was observed; this provides some support for the suggestion that ossification centers first appear in the lower thoracic and upper lumbar regions, and then spread cranio-caudally (30). A similar pattern of microarchitectural change, however, is common to all regions (Figure 6). During gestation (6–9 months), there was a steady increase in BV/TV, peaking at term (Figure 5A). Term vertebrae actually possessed a higher BV/TV than older infant and childhood specimens. In contrast, during infancy (0–1 years), vertebrae exhibited a significant decrease in BV/TV, which dropped by over 2/3rds from 0.310 at term to 0.079 at 1.2 years (Figure 5A). Throughout early childhood (1.2–2.5 years), BV/TV remained constant (Figure 5A).

The observed developmental changes in BV/TV are due to variation in the size and number of trabecular elements. Increasing BV/TV during gestation was solely attributable to increased Tb.N (Figure 5C) because Tb.Th decreased only slightly (Figure 5B). The loss of BV/TV during infancy was due to a reduction in both Tb.Th (Figure 5A) and Tb.N (Figure 5B). During childhood, BV/TV remained constant because Tb.Th increased (Figure 5B) but Tb.N decreased (Figure 5C). Hence, gestation is characterized by increasing Tb.N, whereas infancy and childhood is characterized by a decrease in Tb.N. Further, gestation and infancy are characterized by decreasing Tb.Th whilst childhood is characterized by an increase in Tb.Th.

Developmental changes in trabecular microarchitecture suggest that different bone modeling processes occur during gestation, infancy, and childhood. During gestation, the number of nodes increases (Conn.D Figure 5D), as might be expected, given the increase in Tb.N. The trabeculae became more plate-like (SMI; Figure 5E) suggesting the pores between trabeculae were filled in. Hence, the gestational period seems to be characterized by deposition of bone tissue, the formation of new trabeculae and nodes, as well as the conversion of existing rod-like trabeculae to plates. In contrast during infancy, the number of trabecular nodes decrease (Conn.D; Figure 5D) as a result of decreasing Tb.N. The trabeculae became more rod-like indicating the removal of tissue between trabeculae (SMI; Figure 5E). Hence, trabecular development in the first year is probably defined by resorption of bone tissue, including entire trabeculae and the perforation of plates. Childhood was the only period that was not characterized by a change in trabecular mass because some trabeculae became thicker (Figure 5B) whilst others were removed entirely (Figure 5C). Loss of trabeculae probably caused the apparent reduction in Conn.D (Figure 5D) whilst deposition of tissue may have affected the increase in SMI (Figure 5E) by filling the pores between trabeculae. Thus, childhood development appears to be characterized by selective deposition and resorption of tissue at different sites. Overall then gestational development is characterized by bone deposition, which reverses to resorption during infancy and then gives way to a more balanced pattern of selective deposition and resorption in childhood.

### Mechanisms controlling early bone development

The reversal in growth trajectory (switch from increasing to decreasing BV/TV) after birth could be attributed to a change in the epigenetic mechanisms that control bone growth and development, as well as the loading environment. Uterine bone growth is generally thought to be developmentally preprogramed (31, 32) with some adaptive response to loading (33). However, it is generally assumed that after birth, bone shape is largely influenced by loading (34, 35), probably because parturition marks the beginning of an important functional transition from uterine punching and kicking in the fetal position to habitual weight bearing via sitting, crawling, standing, and walking.

Gestational movements start at about 11 weeks, beginning with sporadic and uncoordinated muscle contractions such as punching and kicking (36–38). More complex movements such as flexion of the limb joints or putting the thumb in the mouth begin at about 5 months and a regular schedule of movement by 6 months of age (39). Yet, vertebral trabeculae do not become more organized (Figure 5F) with strongly orientated trabeculae (Figure 6), as bone often does when adapting to loads [Sensu (35, 40)]. Trabecular tissue became more isotropic during fetal development (Figure 5F). Further, toward the end of gestation, the frequency of movements decreases due to lack of space (41), yet the BV/TV continues to increase toward term. Hence, it seems reasonable to infer that gestational development is programed rather than a response to loading. It has been argued that kicking and punching against the uterine wall (or collisions with other limbs) may represent an intrauterine form of resistance training (7, 42). After birth, the frequency of limb movements increases (43). Yet, the infant’s movements typically occur without much resistance, perhaps putting much smaller loads on the skeleton (44). Thus, the loss of bone observed after birth could be argued to occur, at least in part, because bone becomes more responsive to what are actually reduced mechanical loads. However, this is probably not the case because infant bone resorption resulted in a more anisotropic structure (Figure 5F) with a dominant inferior–superior orientation (axis from bottom to top in the vertical plane; Figure 6). Strongly orientated trabeculae are often referred to as arcades (45) or bundles (46–48), which are typically orientated along the principle strain axis. Ryan and Krovitz (11) reported that the primary arcades in the human proximal femur develop during infancy, with an adult-like pattern appearing at about 2 years. Abel and Macho (46) found that the trabecular bundles in the ilium developed during infancy (<6 months) with the adult-like pattern appearing after 12 years. The authors suggested that the appearance of the main trabecular arcades was an adaptation to developing loads as a result of the ongoing emergence and maturation of gait. The loss of tissue during infancy may be essential for developing a highly orientated structure that can resist loads efficiently with minimal bone mass. This would make sense, as it is easier to take away surplus material than add new tissue, which also provides greater phenotypic plasticity.

The continued development of a highly orientated trabeculae structure in the vertebrae during childhood may be a response to developing postural and locomotor loads (49, 50). The dominant loading direction exerted on the vertebrae by sitting, crawling, bipedal standing, and walking were probably along the inferior–superior axis. This is in the form of muscle contraction (49), weight bearing, and ground reaction forces which travel up and down the vertebral column. The structure adapts (34) in response to an altered loading regime; allowing it to become more efficient at resisting compressive loads along an inferior–superior axis. Additionally, the apparent loss of bone that occurred during the first year of life may have served to supply the growing infant with calcium. During pregnancy, maternal calcium levels are depleted leading to reduced concentrations in breast milk that cannot sustain infant levels (51). The observed loss of vertebral bone during infancy coincides with elevated parathyroid hormone levels in comparison to childhood and adolescence (44, 52, 53). Parathyroid hormone increases bone resorption and release of calcium for mineral homeostasis. Therefore, a developmentally programed increase in BV/TV before birth might serve to create a calcium reservoir for the growing infant. Whilst the change from a rod-like to plate-like structure (Figure 5E) that occurred during gestation would have created a larger surface area more suited to osteoclastic resorption for mineral release.

### Term bone mass is never recovered

The result of childhood bone remodeling was that trabecular structure continued to become more strongly orientated (Figure 5F) along the longitudinal axis (Figure 6). During this time, Tb.Th, DA, SMI, and Conn.D metrics all return toward gestational values however the BV/TV and Tb.N never recover. At 2.5 years, both BV/TV and Tb.N metrics are over 2/3rds lower than at term. Comparison with published adult bone volume data indicate that the bone lost during infancy is never completely replaced (Table 2). At term, the BV/TV is about 0.310 but Hildebrand et al. (54) reported that in a modern population of adults (22–94 years), the BV/TV was 0.040–0.226. Several other studies investigating contemporary older adults (50 + years), all reported that the highest BV/TV value was between 0.130 and 0.180 (Table 2). At term, BV/TV is between 27 and 87% higher than in young or old adults, respectively.

**Table 2:** **Trabecular bone volume fraction in adult lumbar vertebrae**.

Paper	Highest BV/TV value	Visualization method	Dataset number (for vertebra)	Age range of specimens
Eckstein et al. (55)	0.15	3D micro-CT	165	52–99
Amling et al. (56)	0.13	2D histological	12	28–84
Nagale et al. (57)	0.15	3D micro-CT	56	55–98
Thomsen et al. (58)	0.18	2D histological	48	18.5–96.4
Chen et al. (59)	0.18	3D micro-CT	56	57–98
Lui et al. (60)	0.14	3D micro-CT	21	60–88
Hildebrand et al. (54)	0.20	3D micro-CT	52	24–92
Lochmuller et al. (61)	0.15	3D micro-CT	165	52–99

Generally, it is thought that early development is important for bone health, but our data show that the infant period is most important, more so in terms of structure than mass. During this time, bone mass decreases as microarchitecture changes, allowing the bone to adapt to the increased demands of loading during adulthood efficiently. Infant and childhood development could be very important for developing adult-type pattern of trabecular morphology. More developmental data from 2 years to adulthood is required to determine whether this is the case.

## Conclusion

Overall the sequential pattern of trabecular growth development appears to be one of gestational overproduction followed by infant “sculpting” of bone tissue during the first year of life, and then subsequent refinement during early childhood. Similar patterns of development have been described in other parts of the body. During embryological hand development, the finger digits are sculpted from the hand paddle by programed cell death occurring in the cells that are no longer needed (62). During brain development, there is an initial overproduction of tissue, which is then refined on the basis of functional activity (63). The data presented in this study appear to show that early trabecular development follows the same basic pattern of overproduction, constructive regression, and refinement. This process probably serves two functional purposes; gestational overproduction creating a calcium reservoir for mineral homeostasis, whilst sculpting of bone during infancy and childhood releases the calcium and allows greater phenotypic plasticity as bone can adapt to the prevailing loading environment. Hence, in line with current thinking (1, 2), early development could be very important for developing bone strength, but through structural changes in trabecular microarchitecture as opposed to mass.

## Conflict of Interest Statement

The authors declare that the research was conducted in the absence of any commercial or financial relationships that could be construed as a potential conflict of interest.

## Supplementary Material

The Supplementary Material for this article can be found online at http://journal.frontiersin.org/article/10.3389/fendo.2015.00067/abstract

Video 1 **Movie of Figure 6**.
